# Spontaneous intracerebral hemorrhage in CADASIL

**DOI:** 10.1186/1129-2377-14-98

**Published:** 2013-12-17

**Authors:** Lifei Lian, Dujuan Li, Zheng Xue, Qiming Liang, Feng Xu, Huicong Kang, Xiaoyan Liu, Suiqiang Zhu

**Affiliations:** 1 Department of Neurology, Tongji Hospital, Tongji Medical College, Huazhong University of Science and Technology, 1095 Jiefang Ave, Wuhan, Hubei 430030, P.R. China; 2 Department of Pathology, Henan Provincial People’s Hospital, People’s Hospital of Zhengzhou University, No.7 Weiwu Road, Zhengzhou, Henan 450003, P.R. China

**Keywords:** Migraine, CADASIL, Intracerebral hemorrhage, *NOTCH*3, Microbleeds

## Abstract

**Background:**

Cerebral autosomal dominant arteriopathy with subcortical infarcts and leukoencephalopathy (CADASIL) is a rare hereditary small vascular disease and its mainly clinical manifestations are ischemic events. Spontaneous intracerebral hemorrhage (ICH) involvement in patients with CADASIL is extremely uncommon.

**Case report:**

A 46-year-old normotensive Chinese man developed a large hematoma in the left basal ganglia after he was diagnosed with CADASIL 2 months ago, the patient did not take any antithrombotics. Susceptibility weighted imaging at pre-ICH showed multiple cerebral microbleeds (CMBs) in the bilateral basal ganglia. He experienced migraine at about 10 months post-ICH. To our knowledge, this is the first report of ICH in CADASIL patients with Arg90Cys mutation in exon 3.

**Discussion and conclusions:**

ICH should be considered when evaluating new attacks in CADASIL patients. Thus, MRI screening for CMBs might be helpful in predicting the risk of ICH and guiding antithrombotic therapy. In addition, strict control of hypertension and cautious use of antithrombotics may be important in this context.

## Background

Cerebral autosomal dominant arteriopathy with subcortical infarcts and leukoencephalopathy (CADASIL) is a rare hereditary, autosomal dominant, cerebral small vessel disease caused by mutations in the *NOTCH*3 gene [1]. The disease is clinically characterized by migraine, subcortical ischemic events, psychiatric disorders, and cognitive impairment eventually leading to dementia and disability [2,3]. The accumulation of granular osmiophilic material (GOM) in arterial walls on ultrastructural examination is pathognomonic. Typical abnormalities on magnetic resonance imaging (MRI) in CADASIL include severe leukoencephalopathy accentuated in the temporal poles and multiple subcortical lacunar infarcts. CADASIL has been considered a primarily ischemic form of cerebral vascular disorder, spontaneous intracerebral hemorrhage (ICH) in CADASIL is rarely reported. The exact mechanisms of ICH in this setting are unknown currently. Here we report a middle-aged Chinese male with CADASIL who developed a large ICH during the disease process. As far as we know, this is the first report of ICH in patients carrying the Arg90Cys mutation in exon 3 of the *NOTCH*3 gene. We also briefly reviewed the literature on this topic.

## Case presentation

On 22nd March 2012, a 46-year-old right-handed Chinese male, who had been misdiagnosed with multiple sclerosis three years earlier in another hospital because of the white matter lesions in MRI, came into the hospital due to sudden dysarthria and left hemiparesis. His blood pressure was 130/80 mmHg and his temperature was 36.6˚C. A neurological examination revealed left hemiparesis, dysarthria, spasticity in his extremities and diffuse brisk deep tendon reflexes, corresponding to a diagnosis of pure motor stroke [4]. Mini-mental status examination was normal (a score of 28 out of 30).

**Figure 1:**
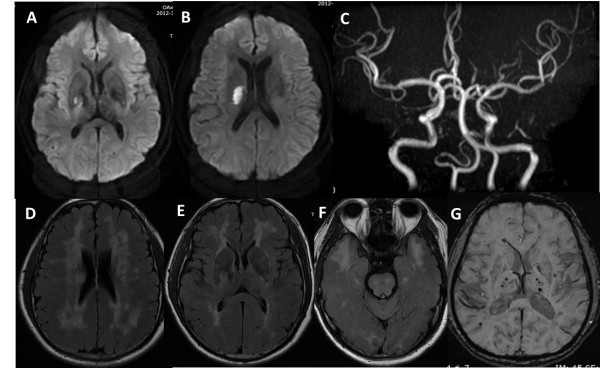
**MRI findings. A-B**. Diffusion-weighted imaging shows an acute cerebral infarct on the right basal ganglia and periventricular areas. **C**. MR angiography reveals no significant stenosis or malformation of major cerebral arteries. **D-F**. FLAIR image demonstrates hyperintensity lesions in the periventricular white matter, basal ganglia, and brainstem, characteristically in the external capsule and anterior temporal pole. **G**. Susceptibility weighted imaging shows multiple cerebral microbleeds in the bilateral basal ganglia.

An acute subcortical infarction was found in the right basal ganglia and periventricular areas on the 2nd day of hospitalization (Figure 1A, 1B). There was no significant stenosis or malformation of major cerebral arteries on MR angiography (Figure 1C). MRI confirmed the severe white matter lesions concentrated around the periventricular, pons, external capsule and the anterior temporal pole and multiple subcortical lacunar infarcts (Figure 1D-1F). Some cerebral microbleeds (CMBs) were also present in the bilateral basal ganglia and subcortical areas (Figure 1G). There was no evidence for head trauma, cavernous hemangioma, arteriovenous malformations, aneurysms or neoplasia. In the past, he did not have any episodes of migraine and any vascular risk factors other than smoking. His family history disclosed that his brother had a similar episode of dysarthria. Blood tests, including a complete blood cell count, glucose, cholesterol, creatinine, erythrocyte sedimentation rate, homocysteine, and C-reactive protein were normal. Hemocoagulation tests were within normal values. Coagulation factors such as factor II, V, VII, VIII, IX and von Willebrand factor were normal. Serological tests for collagen disease were unremarkable, as was the cerebrospinal fluid testing. Lactate and pyruvate levels were normal in blood. Urine analysis was normal with no proteinuria. Cocaine urine test was negative. An echocardiogram was normal, and electrocardiographic monitoring did not show arrhythmias.

**Figure 2:**
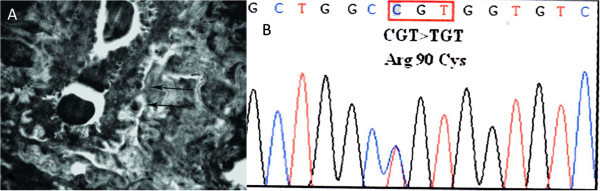
**Ultrastructural examination and *NOTCH*3 gene testing. A**. GOM deposits (arrows) are located in the basement membrane of smooth muscle cells. **B**. Gene analysis indicates a mutation CGT → TGT in codon 90 in exon 3 of the *NOTCH*3 gene.

Since CADASIL was suspected based on the characteristic MRI findings and his family history, a skin biopsy and *NOTCH*3 gene analysis was performed on hospital day 13 after the informed consent. Ultrastructural examination showed the accumulation of GOM in small arterial walls (Figure 2A). *NOTCH*3 gene testing revealed a heterozygote Arg90Cys mutation in exon 3 (Figure 2B). The patient remained normotensive throughout his hospital stay. He was not given any antithrombotics because of gastric hemorrhage 1 year ago. The modified Rankin scale (mRS) score at 30 days after this ictus was 1 point.

**Figure 3:**
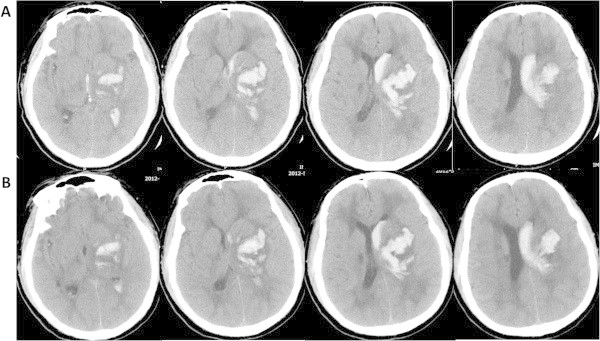
**Head CT scan at the second admission. A**. An acute irregular large hematoma presents near the left basal ganglia with a prominent mass effect, which extended into the ventricular system. **B**. CT scans three days later show no significant change of the size of hematoma.

Two months after discharge, he was admitted again to our hospital with a sudden unresponsiveness. Initial blood pressure was 138/98 mmHg and neurological examination showed mild coma and right spastic hemiplegia. The admission Glasgow Coma Scale score and NIH Stroke scale (NIHSS) score was 11 and 20 respectively. An immediate computed tomography (CT) scan demonstrated an acute ICH in the left basal ganglia extending into the ventricular system (Figure 3A). There was no evidence of trauma on head CT. Follow-up CT three days later showed no significant change of hematoma size (Figure 3B). The patient showed gradual recovery from his right hemiplegia without hematoma evacuation. The 30-day Barthel index and NIHSS score after ICH was 45 and 14 respectively. Then, the patient had an mRS of 2 at 12 months after ICH onset, supporting a favorable outcome. In addition, the patient developed migraine at about 10 months after the second hospital admission.

## Discussion

This study supports the growing evidence for both ischemic and hemorrhagic events in CADASIL, thus expanding its clinical phenotype. MRI screening for CMBs might be helpful to predict the risk of ICH and guide antithrombotic therapy in patients with CADASIL. To our knowledge, this is the first report of ICH in CADASIL patients harboring an Arg90Cys mutation in exon 3 of the *NOTCH*3 gene on chromosome 19.

The development of ICH in patients with CADASIL is extremely rare. As shown in Table 1, there were only 21 reported cases with adequate clinical and genetic data [5-14]. The mean age of these patients was 56.0 ± 13.4 years. Hypertension was present in 16 patients (72.7%). Twelve patients (54.5%) received antithrombotics to prevent ischemic events. ICH was the initial manifestation in 7 patients. The most frequent site of ICH was the basal ganglia. The mean number of CMBs for 14 patients was 19.4 ± 21.8 (range 1 to 85). R544C mutation in exon11 was prevalent (45.0%).

**Table 1:** Clinical, genetic and radiological features of CADASIL patients with ICH

**Author**	**Sex/age**	**Vascular risk factors**	**Symptoms**	**Antithrombotics or statins**	**Location of ICH**	**Gene mutation**		**Number of CMBs**
						**Exon**	**Amino acid**	
Werbrouck [5]	M/45	HT, HC	Migraine, CI, ICH	NA	Basal ganglia	4	R182C	6
Oh [6]	M/39	Smoking, HT, alcohol	CI, ICH	Anticoagulant and statins	Temporal lobe	11	R544C	NA
Sano [7]	M/46	Smoking	CI,Dementia, ICH	Antiplatelet	Putamen	6	A332C	NA
Maclean [8]	M/56	Smoking, alcohol	CI, ICH	Antiplatelet	Frontal lobe	4	R133C	1
Choi [9,18]	F/68	HT	TIA, Dementia, ICH	Antiplatelet (3)	Cerebellum	11	R578C	27
	M/69	HT,DM	Dementia, ICH		Thalamus Parietal lobe	11	R544C	85
	M/61	HT, alcohol	ICH, Headache		Basal ganglia	11	R544C	14
	M/48	HT	ICH, TIA		Basal ganglia	11	R544C	5
	F/86	HT, HC	Headache, ICH Gait difficulty		Thalamus	NA	NA	32
Lee [10]	M/43	HT	Gait difficulty, CI, ICH	Antiplatelet(5)	Thalamus	4	R133C	NA
	F/56	HT	ICH		Thalamus	11	R544C	NA
	M/57	HT	CI, ICH		Temporal lobe	11	R544C	NA
	M/56	HT	CI, ICH		Putamen, Parietal lobe	11	R544C	NA
	M/35	HT	Gait difficulty, ICH		Putamen	11	R544C	NA
Ragoschke-Schumm [11]	F/47	HT	CI, ICH	NA	Cerebellum	NA	NA	25
Pradotto [12]	M/65	NA	Dementia, ICH	Anticoagulant	Frontal lobe	13	A680G	3
Kotorii [13]	F/72	NA	Gait difficulty, CI Dementia, ICH	NA	Temporal lobe	18	G975C	NA
Rinnoci [14]	M/54	HT	ICH	NA	Basal ganglia	22	A1231C	25
	F/67	HT, Smoking	ICH	NA	Basal ganglia	22	A1231C	3
	M/77	HT, Smoking	ICH, Dementia	NA	Basal ganglia	14	A728C	2
	M/39	HC, DM	SAH	NA	Interpeduncular cistern	24	C1298P	25
This	M/46	Smoking	CI,ICH	Statins	Basal ganglia	3	A90C	18

ICH,intracerebral hemorrhage; CMBs,cerebral microbleeds; HT,hypertension; DM,diabetes mellitus; HC,hypercholesterolemia; CI, cerebral infarctionr; TIA, transient ischemic attack; SAH, subarachnoid hemorrhage; NA, not available.

CMBs were frequently detected on MRI in CADASIL patients. Growing evidence showed CMBs may indicate a risk of ICH or even predict the occurrence of ICH [15-17]. CADASIL was characterized by the depositions of GOM in vascular smooth muscle cells, thus making the arteries more bleed-prone. Choi et al. recently found that the CADASIL patients with hypertension had a significantly higher prevalence of cerebral infarction and ICH and significantly greater numbers of CMBs than without hypertension [18]. Both CMBs and ICH in this reported case were located in the basal ganglia, the most frequent site of hypertensive ICH. Thus, the cause of ICH in this patient may be the additional effect of abrupt fluctuation of blood pressure on the fragile arterioles. Strict control of blood pressure may be crucial in such context, particularly in the presence of CMBs.

The onset of ICH seemed to be associated with specific mutation or the use of antithrombotics. The prevalent occurrence of R544C mutation (45.0%) in this analysis potentially suggested an association between ICH and the genetic mutation. However, previous data showed no relation between genotype and clinical phenotype [3]. Recent studies also warned for the increased risk of ICH by taking antithrombotic agents in CADASIL patients [6,9-11]. In this report, more than 54% of the patients received antithrombotic therapy to prevent ischemic events. However, Choi JC et al. recently reported that the previous antiplatelet use had no contribution to the occurrence of ICH in patients with R544C mutation [18]. Thus, the risk of ischemia versus hemorrhage should carefully be weighed when treating CADASIL patients. MRI screening for CMBs may be helpful in this risk stratification [16,17]. Taking into account the high morbidity and mortality of ICH and lack of proven treatment of CADASIL, routine use of aspirin or anticoagulants to prevent recurrent ischemic stroke might be unnecessary. On the contrary, cilostazol may be an alternative to aspirin in such context due to its efficacy and low risk of hemorrhage [19].

We presented a clinical note reporting the case of a middle-aged Chinese man with ICH two months after a pure motor stroke due to acute small subcortical ischemic stroke in CADASIL. The reported patient almost completely improved at 30 days after the first admission. Recently, a clinical study reported that patients with pure motor stroke showed significant improvement with a higher ratio of symptom free at hospital discharge than patients with non-lacunar stroke [4]. In addition, this report might help to elucidate the course of improvement after ICH. The patient showed profound improvement from severe disability at the second hospital discharge to a favorable long-term outcome at 12 months post-ICH. Arboix et al. recently documented that the site of hematoma had an impact on the early outcome in patients with ICH [20]. Thus, whether ICH clinical trials should use a longer duration of follow-up should be considered and may potentially be influenced by the specific site of ICH.

It is very important to separate CADASIL from other potential mimickers such as mitochondrial encephalomyopathy lactic acidosis and stroke-like episodes (MELAS) or Fabry disease. MELAS is associated with a mutation in the mitochondrial tRNA gene. The typical findings in MRI are bilateral, parieto-occipital, transterritorial ischemic lesions. To the best of our knowledge, only 3 patients with ICH were reported among MELAS syndrome [21]. Fabry disease is a rare X-linked inherited disorder of glycosphingolipid metabolism because of deficient or absent lysosomal α-galactosidase A activity [22]. Its classical manifestations include neuropathic pain, skin lesion and gastrointestinal symptoms, progressive renal and cardiac insufficiency and stroke [22]. Females seemed to be more likely to develop a stroke. In addition, the disease was frequently associated with ischemic stroke.

To date, it is yet to be ascertained whether ICH in CADASIL occurred as a disease process associated with genetic mutation or as a result of hypertension and CMBs or whether it was due to the administration of specific antithrombotics. The combination of these factors may be more prone to the onset of ICH. Future studies should investigate the underlying mechanisms of ICH and guide the stroke prevention in patients with CADASIL.

## Conclusions

ICH should be considered when assessing new events in patients with CADASIL. The mechanisms of ICH might result from the combination of genic mutation and vascular risk factors. Taking into account the risk of ICH, MRI screening for CMBs may be helpful for risk stratification. In addition, stringent control of these modified factors, especially hypertention and cautious use of antithrombotics may be pivotal in CADASIL patients.

## Consent

Written informed consent was obtained from the patient for publication of this case report and any accompanying images. A copy of the written consent is available for review by the Editor-in-Chief of this journal.

## Abbreviations

CADASIL: Cerebral autosomal dominant arteriopathy with subcortical infarcts and leukoencephalopathy; ICH: Intracerebral hemorrhage; CMBs: Cerebral microbleeds; GOM: Granular osmiophilic material; MRI: Magnetic resonance imaging; CT: Computed tomography; mRS: Modified rankin scale; NIHSS: National institutes of health stroke scale; MELAS: Mitochondrial encephalomyopathy lactic acidosis and stroke-like episodes.

## Competing interests

The authors declare that they have no competing interests.

## Authors’ contributions

LL and SZ conceived of the study, participated in clinical management of the patient, reviewed the literature on the item and drafted the manuscript. DL participated in the design of the paper and performed the pathologic investigation. QL participated in gene analysis and drafted the manuscript. FX had the first approach with the patient at the ER, made the correct diagnosis and participated in his first clinical management. ZX has contributed in the clinical management of ICH and revised the manuscript. XL and HK performed the neuroradiological investigation and reviewed the literature concerning the technique. All authors read and approved the final manuscript.
